# Virtual Mind-Body Programming for Patients With Cancer During the COVID-19 Pandemic: Qualitative Study

**DOI:** 10.2196/27384

**Published:** 2021-06-08

**Authors:** Nicholas Emard, Kathleen A Lynch, Kevin T Liou, Thomas Atkinson, Angela K Green, Bobby Daly, Kelly Trevino, Jun J Mao

**Affiliations:** 1 Bendheim Integrative Medicine Center Department of Medicine Memorial Sloan Kettering Cancer Center New York, NY United States; 2 Memorial Sloan Kettering Cancer Center New York, NY United States

**Keywords:** cancer, fitness, meditation, stress, COVID-19, qualitative, coping, wellbeing, psychosocial, virtual health

## Abstract

**Background:**

Patients with cancer are particularly vulnerable to stress and anxiety during the COVID-19 pandemic. Social distancing is critical for patients with cancer; however, it can also reduce their access to psychosocial coping resources.

**Objective:**

The aim of this study was to explore patient experiences to generate a model of how virtual mind-body programs can support the psychosocial well-being of patients with cancer.

**Methods:**

We conducted a qualitative study among patients (aged ≥18 years) who participated in a virtual mind-body program offered by a National Cancer Institute–designated Comprehensive Cancer Center during the COVID-19 pandemic. The program consisted of mind-body group therapy sessions of fitness, yoga, tai chi, dance therapy, music therapy, and meditation. Live integrative medicine clinicians held each session via Zoom videoconferencing for 30-45 minutes. In semistructured phone interviews (n=30), patients were asked about their overall impressions and perceptions of the benefits of the sessions, including impacts on stress and anxiety. Interviews were analyzed using grounded theory.

**Results:**

Among the 30 participants (average age 64.5 years, SD 9.36, range 40-80, 29 female), three major themes were identified relating to experiences in the virtual mind-body program: (1) the sessions helped the patients maintain structured routines and motivated them to adhere to healthy behaviors; (2) the sessions enhanced coping with COVID-19-related-stressors, allowing patients to “refocus” and “re-energize”; and (3) the sessions allowed patients to connect, fostering social relationships during a time of isolation. These themes informed the constructs of a novel behavioral-psychological-social coping model for patients with cancer.

**Conclusions:**

Virtual mind-body programming supported patients with cancer during the COVID-19 pandemic through a behavioral-psychological-social coping model by enhancing psychological coping for external stressors, supporting adherence to motivation and health behaviors, and increasing social connection and camaraderie. These programs have potential to address the behavioral, psychological, and social challenges faced by patients with cancer during and beyond the COVID-19 pandemic. The constructs of the conceptual model proposed in this study can inform future interventions to support isolated patients with cancer. Further clinical trials are needed to confirm the specific benefits of virtual mind-body programming for the psychosocial well-being and healthy behaviors of patients with cancer.

## Introduction

The outbreak of SARS-CoV-2, the virus that causes COVID-19, increased the stress levels of many individuals due to the threat of infection, news of overwhelmed healthcare institutions, and disruptions to daily life [1]. Stress levels may be exceptionally high for patients with cancer; social distancing is critical for this population because they are more susceptible to severe illness and mortality due to COVID-19 [2,3]. However, necessary stay-at-home orders and social distancing measures—which restrict access to parks and exercise facilities—have contributed to a worldwide decrease in physical activity [4] and a loss of usual support networks and other potential coping strategies for stress [5,6]. Thus, there is a critical need to address the enormous psychosocial burden of the COVID-19 pandemic for patients with cancer.

Patients with cancer often experience behavioral [7], psychological [8,9], and social [10] challenges, which are associated with worse cancer-related outcomes [11]. During the COVID-19 pandemic, patients have experienced higher levels of mental distress due to concerns regarding access to safe physical activity [12], significant life and health stressors [13], and loneliness [14]. For example, decreased physical activity due to social distancing or lack of adequate equipment can adversely affect the quality of life of patients with cancer and their mental health [12]. A survey (n=555) of women with current or previously diagnosed ovarian cancer showed that 89% reported “significant cancer worry” due to COVID-19 [15]. In Italy, in a prospective evaluation of patients with lymphoma, 75% of patients stated that “their worries had increased during the pandemic,” and over one-third met diagnostic criteria for anxiety and depression [16]. Patients with cancer may also experience “alarmingly high rates of stress” and “extraordinarily high symptom burden,” which necessitate increased vigilance among oncology providers [13]. In the Netherlands, patients with cancer expressed concerns of loneliness and fears of being in the hospital or not seeing their family due to COVID-19 [17]. Programs that address behavioral, psychological, and social stressors while complying with social distancing measures are critically important for supporting the quality of life of patients with cancer during the COVID-19 pandemic.

Mind-body therapies, such as meditation, yoga, and tai chi, have been shown to reduce stress and anxiety in patients with cancer and enhance their quality of life [18,19]. The American Society of Clinical Oncology and the Society for Integrative Oncology recommend mind-body therapies for treating cancer-related anxiety [20,21]. Exercise has also been effective for patients with cancer in combatting anxiety [22], reducing fatigue and pain [23,24], and improving quality of life [25]. Despite these benefits, patients with cancer and survivors may be limited in their ability to participate in these activities, particularly during the COVID-19 pandemic. Further, the pandemic has challenged society to operate virtually to comply with social distancing mandates [26,27], indicating a need for innovative approaches to support individuals affected by cancer.

In response to these concerns, we rapidly implemented a virtual mind-body program through the Integrative Medicine Service (IMS) at a tertiary National Cancer Institute–designated Comprehensive Cancer Center [28]. The program consisted of a series of virtual, synchronous classes offering a variety of rigorously tested mind-body therapies led by an IMS clinical therapist. The objective of this study was to explore patient experiences of the virtual program to generate a model of how virtual mind-body programs can support the psychosocial well-being of patients with cancer.

## Methods

### Therapy Sessions

We conducted virtual mind-body group therapy sessions using the Zoom video conferencing platform [29] beginning on April 1, 2020. Patients were contacted through the cancer center’s patient messaging portal about virtual programs offered during the COVID-19 pandemic. Once registered, patients chose from a variety of weekly classes, which were held 1-4 times per week for 30-45 minutes. A licensed IMS clinician (eg, licensed dance therapist, certified yoga instructor, nurse specialist/physical trainer) with specific expertise in the oncology setting led each session. Patients could choose to participate in as many sessions as they preferred. Activities ranged from more movement-based (fitness, yoga, dance therapy, or tai chi) to meditative (guided meditation, Zen breathing, or listening to music therapy played by a licensed music therapist). All clinicians provided an overview of the session, 25-40 minutes of content, and 5 minutes for feedback and discussion. Because the program was developed in response to the COVID-19 pandemic, we conducted qualitative interviews for quality improvement between April and August 2020. The hospital’s Institutional Review Board approved a retrospective protocol for the analysis of the quality improvement data.

### Qualitative Interview Procedure

At the conclusion of the sessions, clinicians asked for volunteers to provide feedback on the virtual session. An IMS staff member with qualitative research training (NE) contacted the participants and arranged a telephone interview. The interviews lasted 10-35 minutes and followed a semistructured interview guide generated by study team members with content (JJM, KTL) and methodological (KAL, NE) expertise. The interview guide was organized into the following domains: (1) overall impressions, (2) perception of the benefits of the session, including impacts on stress and anxiety, and (3) unmet needs and recommendations for improvement. Consistent with the practice of semistructured interviewing, the interviewer asked flexible probing questions to further explore relevant themes and topics as they emerged. Probes were iteratively developed throughout the study period based on emerging participant feedback and iterative analysis. Interviews were conducted until thematic saturation was obtained [30], transcribed verbatim, and deidentified to ensure patient privacy.

### Interview Sampling Approach

We purposively sampled participants across session types to ensure representative feedback about each modality. However, participants were allowed—and encouraged—to participate in any virtual mind-body sessions; multiple sessions were often discussed during interviews. As this was a grounded theory study, iterative analysis of transcripts informed our subsequent sampling approach. As we aimed to create a generalized model of how virtual mind-body programs can support the psychosocial well-being of patients with cancer, we sought theoretical saturation across subgroups (ie, saturation across movement-based and meditation-based sessions). Ultimately, the three constructs reported in this manuscript were explored and refined based on participant experiences across integrative medicine (IM) modalities.

### Qualitative Analysis

Two trained qualitative researchers (KAL, NE) independently coded transcripts using a grounded theory approach to facilitate the development of a coping model [31]. The transcripts were first coded in their entirety using an open coding process, wherein the coding team highlighted significant statements and assigned a descriptive or interpretive label. Through consensus meetings, the team refined common labels (eg, impacts on stress and anxiety, social isolation) into codes. This process was facilitated by analytic memo-writing: during this open-coding phase, researchers wrote margin notes on inductively emerging patterns, which were turned into codes. Then, the codebook was solidified and applied across all transcripts, data were compared against the codes (focused coding), and discrepancies in coding were resolved via consensus. Once all transcripts were coded, the lead coder (KAL) reviewed the data to ensure that all significant statements had been assigned a label. Then, the team completed a process of axial coding in which coded statements were condensed into categories, compared, and grouped under thematic labels supported by the text, thereby grounding each category in the data. The code categories were reviewed and refined via group consensus meetings (eg, “impact on life in quarantine” “impact on routines”). To identify the final constructs of the model, the researchers completed a selective coding phase, wherein statements housed within each category where recoded to identify primary themes. Each category was iteratively revisited to identify instances of theoretical saturation, defined as the point at which no new relevant data, coding, or themes emerge. During this phase, analytic memos were used to refine our theoretical categories. After reviewing a code category, researchers independently wrote memos on the key theoretical implications (eg, “socialization as a means of coping”), which were discussed in consensus meetings. To achieve consensus on the final model, coders met with team members who have expertise in IM delivery (JJM, KTL) and refined the constructs as needed. The qualitative software NVivo Pro 12.0 (QSR International) [32] was used to facilitate the analysis and store the final codebook.

## Results

A total of 30 patients participated in qualitative interviews to achieve thematic saturation (Table 1). The majority of the sample was female (29/30, 97%) and White (25/30, 83%), with an average age of 64.5 years (SD 9.36). Participants had various tumor types; breast cancer was the most common (11/30, 37%). Some participants were in active treatment, while others were in survivorship. Although the interviews focused on participant experiences with a single mind-body modality, the majority of participants participated in multiple sessions. Fitness (22/30, 73%), yoga (17/30, 57%), and tai chi (16/30, 53%) were the most popular modalities, which is supported by the authors’ previous publication regarding the feasibility and acceptability of virtual mind-body programs [28].

**Figure 1:**
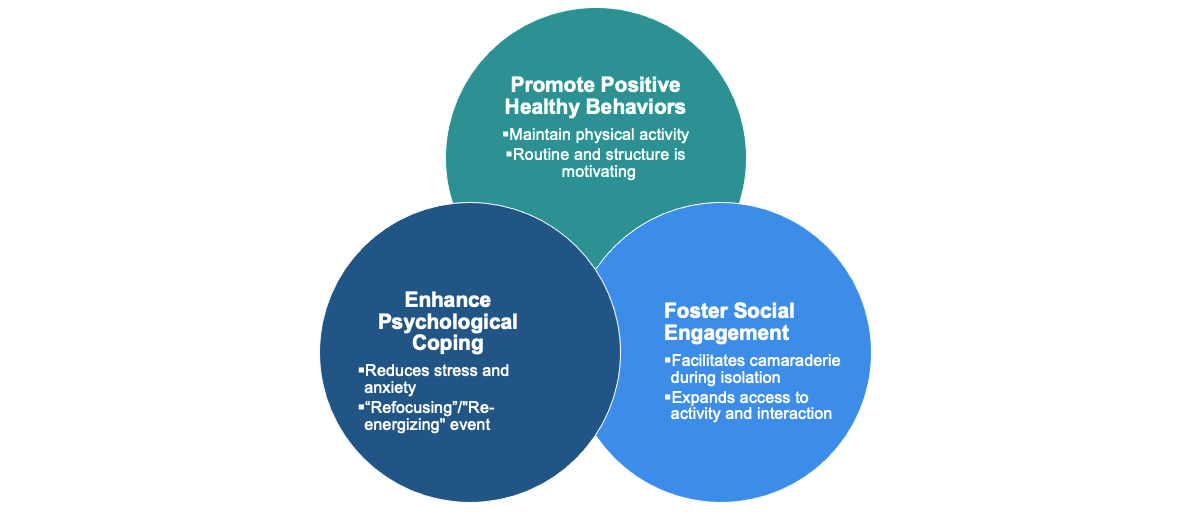
Behavioral-Psychological-Social Coping model of how virtual mind-body programs can support the psychosocial well-being of cancer patients.

Grounded theory analysis identified three major themes related to participant experiences in the virtual mind-body program. These themes indicated that the program (1) promoted positive health behaviors, (2) enhanced psychological coping, and (3) fostered social engagement. Taken together, these three themes informed the constructs of our behavioral-psychological-social coping model, which proposes ways in which virtual mind-body programs can support patients with cancer (Figure 1). Each of these themes and their resulting constructs are explored in detail below (summarized in Table 2).

**Table 1:** Participant demographics (N=30).

Characteristic		Value
Mean age (years) (SD, range)		64.5 (9.36, 40.0-80.0)
**Sex, n (%)**		
	Male	1 (3)
	Female	29 (97)
**Race, n (%)^a^**		
	White	25 (83)
	Asian	4 (13)
	Black	1 (3)
**Cancer type, n (%)^b^**		
	Breast	11 (37)
	Ovarian	4 (13)
	Lung	3 (10)
	Uterine	2 (7)
	Lymphoma	2 (7)
	Tongue	2 (7)
	Colon	1 (3)
	Bladder	1 (3)
	Liver	1 (3)
	Pancreatic	1 (3)
	Prostate	1 (3)
	Skin	1 (3)
**Primary class attendance (interview focus), n (%)**		
	Fitness	5 (17)
	Dance	5 (17)
	Guided meditation	5 (17)
	Music	5 (17)
	Yoga	5 (17)
	Tai chi	3 (10)
	Zen breathing	2 (7)
**Overall class attendance, n (%)**		
	Fitness	22 (73)
	Yoga	17 (57)
	Tai chi	16 (53)
	Dance	14 (47)
	Guided meditation	12 (40)
	Music	13 (43)
	Zen breathing	9 (30)

^a^ Percentage for race adds up to (99%) because percentages were rounded.

^b^ Percentage for cancer type adds up to (99%) because percentages were rounded.

**Table 2:** Major theoretical constructs and associated coding.

Construct	Code category	Major codes
Health behaviors	Impacts on daily life	Maintain movement or routine Behavior change Positive motivation and inspiration
Psychological coping	Impacts on stress and anxiety	Impact on stress and anxiety Effect on mood “Distraction” from other stressors
Social engagement	Perception of benefit	Social camaraderie Reduced isolation Web-based access

### Promotion of Positive Health Behaviors

As people worldwide began to practice social distancing during the COVID-19 pandemic, appointments and social events were canceled, leaving the study participants with a large amount of unstructured time. Participating in the virtual sessions became a way to retain a sense of daily routine, as one music participant described:

[The classes] give my day structure. I look forward to it. I can sort of plan [my day]. I'm home and I'm cleaning, [and] I can have that to look forward to.

This sense of routine became a way to cope with COVID-19-related stress. Patients described the virtual programming as “something to look forward to” amid an uncertain future. Regularly scheduled virtual live classes also motivated participants to engage with health behaviors while social distancing. Discussing their struggle with home-based exercise, a tai chi participant stated:

Yeah, it's great because I try to do exercises on my own, but it's not regular. And, this kind of keeps me regular. Because, it's like “oh it's 1:30, I have to do Tai Chi” and it's kind of like an incentive or motivation. And, especially when there's someone on the other side encouraging you to move or that you can do it.

An option for virtual classes was especially welcome to patients who had been physically active prior to the COVID-19 pandemic. The classes became a way to maintain physical activity and movement when other options for exercise were suddenly unavailable:

But these classes are really, really excellent. And help you in confined spaces and need to get some exercise, you know? […] Before, I used to swim every day and walk in and ride my bike all over the city. You know, it's... now I don't have that.

As a population vulnerable to infection, patients with cancer feel extra pressure to adhere to social distancing guidelines. As the fitness participant describes above, this results in long periods of time in “confined spaces.” Some participants, fearful of infection, described barely leaving their homes while COVID-19 cases peaked:

[I]n March and April I didn't go out much and I wasn't even walking, which is my one major, you know, exercise. So... getting back to movement and moving throughout the day at whatever time, you know, it's really helpful.

For these participants, the virtual classes created a structure to engage in physical activity, promoting positive health behaviors without placing participants at risk of exposure.

### Enhancement of Psychological Coping

Across interviews, participants expressed that the virtual classes enabled them to cope with the daily uncertainties of the COVID-19 pandemic. Feeling inundated with new and often-conflicting information, patients found the sessions to be a grounding experience. As one music participant described:

I think as a patient, lots of times the problem is that you have all these kind of racing thoughts […] you feel quite like engaged in these classes. Like it can really make you focus on the current session.

Participants of the meditative sessions—music, Zen breathing, and guided meditation—found the virtual sessions to be “refocusing” events. By concentrating on their breathing and sensory experiences (ie, calming sounds, visualizations), patients felt that they were able to redirect their attention from external stressors, as one meditation participant stated:

I think I have less stress after the session. And it... because it's calming and it's refocusing, and it helps with channeling the energies in a different place.

Similarly, participants of the movement-based sessions—fitness, dance, yoga, and tai chi—perceived a reduction in stress due to the virtual sessions. For these participants, the virtual sessions were a “re-energizing” event, presenting a distraction from COVID-19–related anxieties. In the words of a dance participant:

It's almost like taking a shower you [are] getting rejuvenated. Now, you can recreate the whole world, right? You sit down, you're nice and calm and your body feels like you're relaxed and you don't have a care in the world about what's going on around you.

Additionally, one participant discussed that yoga classes were particularly helpful after the abrupt transition to isolation:

It [the class] helps, it really helps. Especially in the beginning when all this started, everybody was so stressed. This has helped me a lot to keep my mind out of all this stuff […] now it’s a little easier, but it still really helps me.

Both movement- and meditative-based sessions became ways for the participants to cope with the uncertainties surrounding COVID-19, particularly in the context of cancer care. As a result, participants across interviews described feeling both less anxious and less stressed after the classes:

I'm feel[ing] less stressed, I feel relaxed, I feel energized and that continues afterward. I mean, I find it really, it helps. It really helps dealing with the stress and anxiety of this whole quarantine time.

### Fostering Social Engagement

Cancer can be socially isolating, and participants perceived this isolation to be amplified during the COVID-19 pandemic. As one music participant described, the upheavals created by the pandemic caused her to feel cut off—socially and physically—from friends and loved ones:

[Y]ou feel really alone. Fortunately, I have my husband so at least I'm not like completely alone, but still that's the only way you get to connect with other people. Some of my friends, they are really busy with kids at home, and with working from home... yeah, somehow, they actually turned out to be even busier than before. So sometimes I don't feel very...like I don't want to bother them all the time.

As she went on to describe, the virtual sessions became a way to connect with friends she had met during previous in-person classes:

So, this [class] keeps you feeling you're connected to the community and some fellow patients you happen to see are attending the same session, and it was like, “Wow, it's you” and all this and then we start texting each other. Sometimes I receive these surprise texts is like, “Oh, is it you in the session?” And it just feels... like you need some excitement and surprises, like once in a while. So this provides like a platform for people to continue interacting that way.

Participants discussed the benefit of seeing other patients with cancer and survivors via virtual classes. Faced with unique stressors and concerns during the COVID-19 pandemic, the participants found it comforting to connect with other participants going through a similar experience. As one yoga participant stated, the classes helped her feel “less alone”:

It's helped me to calm down and feel like you're not alone. There are other people doing it and going through this also.

A few participants also stated that virtual programming enabled them to engage in mind-body group sessions for the first time. These participants were unable to attend the pre–COVID-19 in-person classes at the hospital due to geographic, time, or mobility constraints, as in the case of one dance participant:

I'm on oxygen. And if this was anything that was done at the Integrative Medicine Center, I would never go—because of the location. So, you know, this gives me a chance to participate.

Therefore, in addition to enabling isolated patients to virtually reconnect, virtual programming enabled other patients to engage for the first time.

### The Behavioral-Psychological-Social Coping Model

The three major themes identified in our analysis informed our coping model (Figure 1). Virtual mind-body programs have the potential to support patients with cancer in three interrelated ways. (1) Regularly scheduled classes motivate participants to maintain positive health behaviors and create a sense of structure and routine. (2) Through accessing therapies that allow patients to “refocus” and “re-energize,” participants can enhance their ability to psychologically cope with external stressors. (3) Virtual, synchronous sessions, which enable participants to see and communicate with one another, facilitate social connection and camaraderie during a time of isolation and expand access to vulnerable individuals. Taken together, these three constructs provide a model for how virtual mind-body programming can support psychosocial well-being among patients with cancer.

## Discussion

### Principal Findings

The COVID-19 pandemic disproportionately impacts the psychosocial well-being of patients with cancer and survivors due to the unique stressors they encounter as a result of public health measures. This study identifies the constructs of how virtual mind-body services can promote healthy behaviors, enhance psychological coping, and facilitate social connections for patients with cancer during the COVID-19 pandemic and potentially beyond, particularly for patients with limited physical access to the IMS. These qualitative themes form the basis of a behavioral-psychological-social coping model informing how virtual mind-body services can be an accessible and scalable way to address patients' psychosocial challenges.

Our study adds to emerging literature regarding how virtual tools, such as virtual mind-body programming, can address psychological symptoms patients with cancer face during the COVID-19 pandemic. Avancini et al [33] encouraged the use of telehealth and virtual programs for at-home exercises to increase social support and adhere to exercise guidelines. A review of web-based interventions to address the psychosocial needs of patients with cancer demonstrated “promise” in addressing pain, depression, and quality of life measures [34]. Additionally, access to clinicians through virtual web-based visits and telehealth can address existing barriers to care and has potential to fill important gaps in quality cancer care [35]. Our study builds on this literature by providing a model for how virtual mind-body programs can benefit patients with cancer. As a result of the COVID-19 pandemic, we have implemented a virtual tool that specifically identifies points of intervention for maintaining healthy behaviors, addressing psychological issues, and enhancing social connection among isolated patients with cancer.

Research on the benefits of mind-body programs for patients with cancer has focused on mindfulness through the individual use of apps or websites [36-38]. Previous mindfulness interventions are either nonsynchronous (ie, prerecorded, with no opportunities for real-time interaction) or focused on a specific cancer type. The intervention described in this study overcomes barriers to in-person delivery while offering packaged mind-body therapies to patients with all cancer types. Additionally, the program provided multiple modalities, offering patients a choice and a sense of control in selecting programs that met their needs and preferences. Real-time participant interaction with IMS clinicians and fellow patients can help treat loneliness and increase social interaction, which can, in turn, reduce the uncertainty and stressors faced by many patients with cancer [11]. The constructs of the coping model proposed in this study can inform future interventions to support isolated patients with cancer, even after the COVID-19 pandemic.

The virtual mind-body program in this study used an accessible pre-existing video conferencing platform to disseminate therapeutic modalities to patients. By using existing technologies, this program offers a more scalable model for adapting services from in-person to virtual. Although apps or software may be appealing, the costs associated with their development and maintenance may not be sustainable for all programs [39]. According to the Pew Research Center, approximately three quarters of Americans have broadband high-speed internet access at home, and a growing number use their smartphones to access the internet [40]. Thus, an internet-based program using a pre-existing user-friendly platform may provide an accessible and sustainable alternative to in-person services for patients and their providers.

Although the profound isolation associated with the COVID-19 pandemic is a unique experience, barriers to accessing IM, loneliness, and mental distress are common among patients with cancer; the virtual mind-body program has potential to provide benefits well beyond the current pandemic. The behavioral-psychological-social coping model proposed in this study complements other biopsychosocial frameworks [41,42] by focusing on the behavioral, psychological, and social aspects of virtual mind-body programs while being attentive to the unique experiences and challenges of patients with cancer. This model can be used to guide the development and evaluation of future virtual mind-body programs and can provide a structure for addressing the interconnected issues that patients with cancer face. Future application of this model may benefit clinical services and research by providing a multifaceted and patient-informed mechanism of coping with psychological distress.

### Limitations

This study has a number of limitations. The sample is primarily female and White due to the nature of the voluntary interviews, and the results may not be generalizable to other populations. Although we interviewed patients with a range of cancer types, patients with breast cancer were overrepresented in this sample. Further, virtual classes may not be accessible to all patients due to limited internet or technology access, such as patients in rural areas with low bandwidth. Data on Medicare telemedicine reimbursements suggests that virtual access disparities are especially prominent in patients with lower socioeconomic status, who are older than 85 years, and who are in communities of color [43]. Additionally, mind-body classes were provided in the context of clinical care rather than in a controlled research project with participants who self-selected to participate. Therefore, the themes are based on the perspectives of patients and survivors who participated in the program and agreed to complete an interview. Differentiating programs for patients on active treatment and those in survivorship phases can further guide future interventions.

### Conclusion

This study identified how virtual mind-body programs can support adherence to health behaviors, enhance psychological coping with external stressors, and increase social connection and camaraderie when in-person services are not accessible. The COVID-19 pandemic provided an opportunity to better understand the broader experiences of isolated patients with cancer, enabling us to identify critical points of intervention. The virtual mind-body model proposed here has the potential to support patients with cancer to address the behavioral, psychological, and social challenges that they face during and beyond the COVID-19 pandemic.

## Abbreviations

IM: integrative medicine
IMS: Integrative Medicine Service
